# Correlation-Driven Topological Transition in Janus Two-Dimensional Vanadates

**DOI:** 10.3390/ma16041649

**Published:** 2023-02-16

**Authors:** Ghulam Hussain, Amar Fakhredine, Rajibul Islam, Raghottam M. Sattigeri, Carmine Autieri, Giuseppe Cuono

**Affiliations:** 1 International Research Centre MagTop, Institute of Physics, Polish Academy of Sciences, Aleja Lotników 32/46, 02668 Warsaw, Poland; 2 Institute of Physics, Polish Academy of Sciences, Aleja Lotników 32/46, 02668 Warsaw, Poland

**Keywords:** correlation-driven topological transition, vanadates, density functional theory, 2D ferromagnetism

## Abstract

The appearance of intrinsic ferromagnetism in 2D materials opens the possibility of investigating the interplay between magnetism and topology. The magnetic anisotropy energy (MAE) describing the easy axis for magnetization in a particular direction is an important yardstick for nanoscale applications. Here, the first-principles approach is used to investigate the electronic band structures, the strain dependence of MAE in pristine VSi_2_Z_4_ (Z = P, As) and its Janus phase VSiGeP_2_As_2_ and the evolution of the topology as a function of the Coulomb interaction. In the Janus phase the compound presents a breaking of the mirror symmetry, which is equivalent to having an electric field, and the system can be piezoelectric. It is revealed that all three monolayers exhibit ferromagnetic ground state ordering, which is robust even under biaxial strains. A large value of coupling J is obtained, and this, together with the magnetocrystalline anisotropy, will produce a large critical temperature. We found an out-of-plane (in-plane) magnetization for VSi_2_P_4_ (VSi_2_As_4_), and an in-plane magnetization for VSiGeP_2_As_2_. Furthermore, we observed a correlation-driven topological transition in the Janus VSiGeP_2_As_2_. Our analysis of these emerging pristine and Janus-phased magnetic semiconductors opens prospects for studying the interplay between magnetism and topology in two-dimensional materials.

## 1. Introduction

Since the observation of intrinsic ferromagnetism in two-dimensional layered materials (2D) such as CrGeTe_3_ [1] and CrI_3_ [2], the fields of magnetism and spintronics have received tremendous research attention in the 2D limit [3,4,5,6,7,8,9,10,11,12,13,14]. The atomically thin 2D magnetic materials are considered ideal systems, where the magnetic and spin-related features can effectively be controlled and modulated via proximity effects, electric field, magnetic field, strain, defects and optical doping [15,16,17,18,19,20,21,22]. Unlike bulk materials, where magnetic ordering is possible without magnetic anisotropy, long-range magnetic ordering in layered 2D materials is not conceivable in systems deprived of magnetic anisotropy, which is necessary to balance out thermal fluctuations [23]. Due to the fact that magnetic anisotropy is primarily caused by spin-orbit coupling (SOC) effects [24], SOC becomes a crucial characteristic. Furthermore, spintronic devices such as magnetic tunnel junctions and spin valves show enhanced performance based on 2D magnetic structures with substantial magnetic anisotropy [25,26,27]. It has been demonstrated that strain engineering is an effective method of tuning the magnetic, electronic and optical characteristics of materials [28,29,30,31,32,33].

The recently discovered new family of 2D layered materials MA_2_Z_4_, where M, A and Z represent the transition metal atoms (Mo, W, Hf, Cr, V), IV-elements (Si, Ge) and V-elements (N, As, P), respectively [34], has sparked intense interest in different studies [35,36,37,38,39,40,41,42,43,44]. These layered materials exhibit outstanding mechanical, electronic, magnetic and optical properties [35,38,44,45,46,47,48,49,50,51,52,53,54,55,56,57]. It was shown that in the Janus phases of these compounds, the breaking of the mirror symmetry brings Rashba-type spin-splitting [58,59,60,61] and that this, together with the large valley splitting, can give an important contribution to semiconductor valleytronics and spintronics. In the present work, the structural, electronic and magnetic properties of pristine VSi_2_Z_4_ (Z = P, As) and their Janus phase VSiGeP_2_As_2_ are explored. We found ferromagnetic ordering in these systems, and their magnetic anisotropy energy (MAE) reveals a strong dependency on the biaxial strain. In addition, an out-of-plane direction is found as an easy axis for the magnetization of VSi_2_P_4_, while an in-plane direction is favored in VSi_2_As_4_ and VSiGeP_2_As_2_. In the Janus phase, the compound presents breaking of the mirror symmetry. This can give piezoelectric properties, and is equivalent to having an electric field, which can manipulate magnetism and produce skyrmions in 2D materials [62,63]. Intriguingly, there occurs a topological phase transition from a trivial to topologically non-trivial state in VSiGeP_2_As_2_ monolayer, when the Hubbard U parameter is increased. Our investigation of these compounds opens prospects for studying their intrinsic magnetism, the interplay between magnetism and topology in two-dimensional materials and spin control in spintronics.

## 2. Computational Details

A first-principles relativistic approach based on density functional theory (DFT) using the Vienna Ab Initio Simulation Package (VASP) [64,65] is employed. The Perdew–Burke–Ernzerhof (PBE) formalism in the framework of generalized gradient approximation (GGA) is used to include the electron exchange-correlation [66]. Also, the projector-augmented wave scheme is implemented to resolve the Kohn-Sham equations through the plane-wave basis set. An energy cutoff of 500 eV is considered for the expansion of wave functions. The Monkhorst–Pack scheme is applied for *k*-point sampling with 15 × 15 × 1 *k*-point mesh. The lattice constants were optimized at the PBE level. The optimized lattice constant for the Janus VSiGeP_2_As_2_ structure is 3.562 Å, which is between those of VSi_2_P_4_ (3.448 Å) and VSi_2_As_4_ (3.592 Å) monolayers. In addition, the convergence criterion for force is taken as 0.0001 eV/Å, while 10^−7^ eV of energy tolerance is considered for the lattice relaxation. Also, the number of electrons treated as valence is 41. In examining the dynamical stability, a 4 × 4 × 1 supercell of VSiGeP_2_As_2_ monolayer is taken for calculating the phonon dispersion using the PHONOPY code [67]. The GGA + U routine, along with SOC, is executed, and the strongly correlated correction intended for V-*3d* is considered throughout the calculations. The values of the Hubbard parameter used for the d-orbitals of V are U = 4 eV for VSi_2_P_4_, and 2 eV for VSi_2_As_4_ and VSiGeP_2_As_2_, and the Hund coupling J_H_ is set at 0.87 eV. The main source of SOC in this compound is As; the value of SOC for As is estimated to be 0.164 eV [68,69].

## 3. Results and Discussion

**Figure 1:**
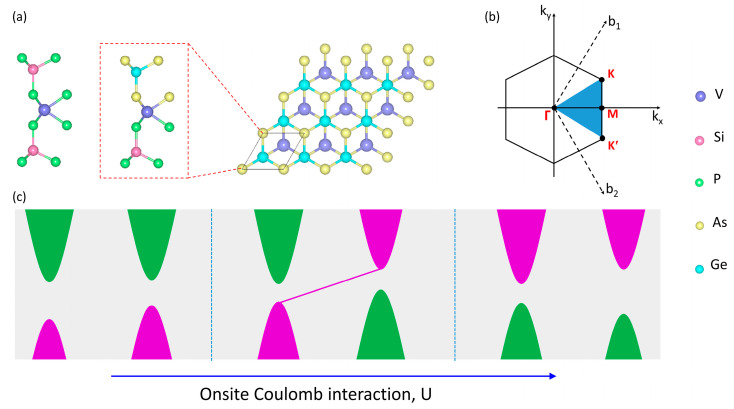
(**a**) Side view of VSi_2_P_4_ monolayer, side and top views for Janus phase VSiGeP_2_As_2_ primitive cell. (**b**) 2D Brillouin zone with the high-symmetry points indicated by red letters. (**c**) Schematic representation for the topological phase transition as a function of onsite Coulomb interaction observed in VSiGeP_2_As_2_ monolayer.

The monolayered VSi_2_Z_4_ (Z = P, As) 2D materials crystallize in a hexagonal geometry with P$\bar{6}$m2 (No. 187) as the space group. These structures are seven-atom thick monolayered systems; the atoms are strongly bonded together with the order as Z-Si-Z-V-Z-Si-Z for pristine and P-Si-P-V-As-Ge-As in the case of the Janus phase. Figure 1a shows the pristine VSi_2_P_4_, VSi_2_As_4_ and Janus VSiGeP_2_As_2_ structures. The VSi_2_Z_4_ (Z = P, As) monolayers have broken inversion symmetry while protecting the mirror-plane symmetry with respect to V plane. In addition, the primitive cell with side and top views is shown for the Janus VSiGeP_2_As_2_ phase in Figure 1a, which presents the breaking of mirror symmetry with regard to the V atom. This is equivalent to an electric field, and the system can show piezoelectricity. The optimized lattice constants for VSi_2_P_4_ and VSi_2_As_4_ monolayers are 3.448 Å and 3.592 Å, respectively, whereas, for the Janus VSiGeP_2_As_2_ structure, it is 3.562 Å. Figure 1b presents the 2D Brillouin zone with the high-symmetry points indicated by red letters. Figure 1c shows the schematic representation for the topological transition as a function of onsite Coulomb interaction in VSiGeP_2_As_2_ monolayer.

**Figure 2:**
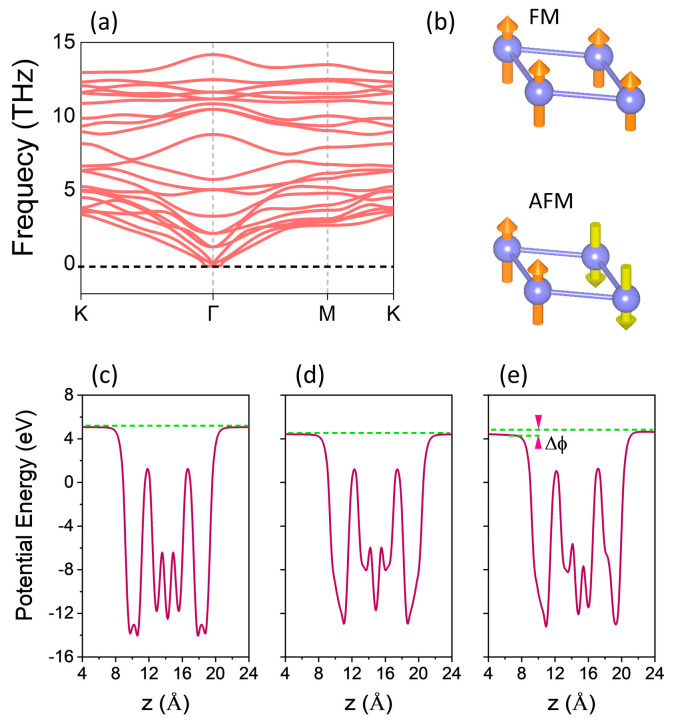
(**a**) The phonon dispersion for the Janus VSiGeP_2_As_2_ monolayer indicating no imaginary frequencies. (**b**) Two magnetic configurations FM and AFM, considered to evaluate the magnetic ground state. The planar average electrostatic potential energy of (**c**) VSi_2_P_4_, (**d**) VSi_2_As_4_, and (**e**) Janus VSiGeP_2_As_2_ monolayers. The work function difference ΔΦ is estimated to be 0.35 eV for the Janus phase.

The stabilities of pristine VSi_2_Z_4_ (Z = P, As) monolayers and the Janus VSiGeP_2_As_2_ structure were studied through the cohesive energies and the phonon dispersion. The cohesive energies per atom (*E*_c_) were computed; for VSi_2_Z_4_, *E*_c_ = [*E*_VSi2Z4_ − (*E*_V_ + 2*E*_Si_ + 4*E*_Z_)]/7, where the energy terms *E*_VSi2Z4_, *E*_V_, *E*_Si_, *E*_Z_ represent the total energies of the VSi_2_Z_2_ monolayer and that of V, Si and *Z* atoms, respectively. Similarly, for the Janus VSiGeP_2_As_2_, it can be written as *E*_c_ = [*E*_VSiGeP2As2_ − (*E*_V_ + *E*_Si_ + *E*_Ge_ + 2*E*_P_ + 2*E*_As_)]/7. The values of E_c_ were calculated as −3.25, −2.60 and −2.92 eV/atom for VSi_2_P_4_, VSi_2_As_4_ and VSiGeP_2_As_2_. These are relatively high compared to recently reported MoSiGeP_2_As_2_ (−2.77 eV/atom), WGeSiP_2_As_2_ (−2.84) [61] and other transition-metal based 2D Janus materials such as MoSSe, WSSe (−2.34 eV, −2.06 eV) [70]. Here, the phonon dispersion for VSiGeP_2_As_2_ is calculated along the high symmetry directions of the Brillouin zone (K-Г-M-K) with the method of finite difference implemented in the Phonopy code. Figure 2a shows the phonon dispersion of VSiGeP_2_As_2_ revealing no imaginary frequency modes, thus dynamically stable. The pristine monolayers VSi_2_Z_4_ (Z = P, As) are already reported to be dynamically stable [9,31]. The large values of cohesive energies E_c_, and the dynamical stability established from phononic spectra, can promise their experimental realization. 

The electronic configuration for an unbonded V atom is 3d^3^4s^2^. However, the V atom in VSi_2_Z_4_ (Z = P, As) is trigonal-prismatically coordinated with six Z atoms. This type of crystal field divides the *3d* orbitals into *dz^2^, d_yz_/d_xz_* and *d_xy_/d_x_^2^_−y_^2^*, as reported in MoS_2_ for Mo atoms, which requires that *dz^2^* orbital should be occupied first [71]. The V atom donates four electrons to neighboring Z atoms, with one electron remaining, giving rise to V^4+^ valence state. With this one unpaired electron in *dz^2^*, a magnetic moment of 1 μ_B_ is expected according to Hund’s rule and the Pauli exclusion principle. Our DFT calculations indeed revealed a magnetic moment of ~1 μ_B_ per formula unit for VSi_2_Z_4_ (Z = P, As) and Janus VSiGeP_2_As_2_ structures. In addition, the total energies of two distinct magnetic configurations were evaluated in order to determine the magnetic ground state. For the antiferromagnetic (AFM) configuration, the magnetic moments were made antiparallel to nearest neighbors, while all of the magnetic moments were initialized in the same direction in the ferromagnetic (FM) configuration. In both instances, the spin orientations were off-plane. Figure 2b depicts these two common magnetic orderings with a 2 × 2 × 1 supercell, for which the total energies and magnetic moments of the FM and AFM configurations were calculated, respectively. For the 2 × 2 × 1 supercell, a magnetic moment of ~4.0 μ_B_ is revealed for both the pristine and Janus phases in the FM state, while 0 μ_B_ is observed with the AFM alignment. Moreover, the energy difference between the FM and AFM states (E_FM_ − E_AFM_) indicated negative energies, strongly suggesting intrinsic ferromagnetism in VSi_2_Z_4_ (Z = P, As) monolayers and their Janus structure. The optimized lattice constants a_o_, the energy difference between the FM and AFM alignments and the easy axis for the magnetization for VSi_2_Z_4_ (Z = P, As) and Janus phase are reported in Table 1. We also computed the average electrostatic potential profiles along the *z*-axis for the pristine and the Janus phase. As indicated in Figure 2c,d, the profiles are symmetric for VSi_2_Z_4_ (Z = P, As). However, in the case of Janus VSiGeP_2_As_2_, the calculated average electrostatic potential is rather asymmetric with a work function difference, ΔΦ of 0.35 eV (Figure 2e).

**Figure 3:**
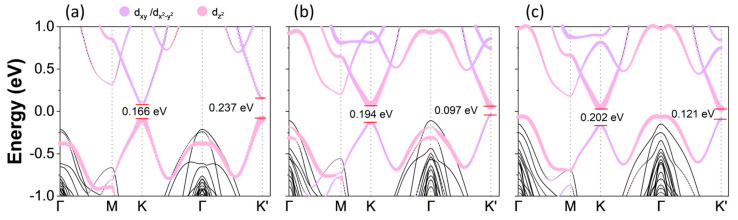
Orbitally resolved electronic band structures of (**a**) VSi_2_P_4_, (**b**) VSi_2_As_4_ and (**c**) VSiGeP_2_As_2_ Janus structure. The V-*3d* orbitals are represented by different colors, where the size of the colored dot describes the contribution from particular orbitals. The contribution decreases as the size of the colored dot decreases. The values of the Hubbard parameter used for the d-orbitals of V are U = 4 eV for VSi_2_P_4_, and 2 eV for VSi_2_As_4_ and VSiGeP_2_As_2_.

The transition metal based 2D materials host degenerate energy valleys (at the K/K′ points of Brillouin zone) owing to a lack of inversion symmetry. Such energy valleys can be manipulated and utilized in valley-spin Hall effects and valley-spin locking [72,73,74]. Generating and controlling the valley polarization by making the K/K′ valleys non-degenerate is a big challenge in valleytronics. There are multiple means to lift this valley degeneracy between the K/K′ valleys and consequently generate the valley polarization. However, when an external magnetic field is removed, the polarization disappears. In general, the 2D monolayers preserve the long-range ferromagnetic ordering due to the intrinsic anisotropy. Specifically, in V-based TMDs, the spontaneous valley polarization results from the magnetic interaction among the V-3d electrons, which is independent of external fields and enables the modulation of spin and valley degrees of freedom. We therefore investigated the orbital-projected band structures of VSi_2_Z_4_ (Z = P, As) and Janus VSiGeP_2_As_2_ monolayers, as shown in Figure 3. As illustrated, all three structures reveal nondegenerate energy values at the K and K′ valleys, and as a result they show different energy band gaps at the two valleys. The valley polarization is defined as [5], ΔE_v/c_ = E^K′^_v/c_ − E^K^_v/c_, where E^K,K′^_v/c_ represents the energies of electronic band edges at K/K′ valleys, correspondingly. In the case of VSi_2_P_4_, using this definition, we found a valley polarization of 76.6 meV in the bottom conduction band, while the top valence bands at K/K′ valleys remain almost degenerate with valley polarization of −3.9 meV. By contrast, for VSi_2_As_4_, the valley polarization is −8.2 meV in the bottom conduction band, whereas that of the top valence band is calculated to be ~88 meV. On the other hand, in the Janus phase, the bottom conduction bands at K/K′ remain almost degenerate in energy with valley polarization of −5 meV and 73.3 meV in the top valence bands. This reveals that intrinsic ferromagnetism is much more efficient in creating valley polarization. In addition, the conduction band minimum (CBM) in VSi_2_P_4_ is composed of V-*d_xy_* and V-*d_x_^2^_−y_^2^* states at both K and K′ points, while the valence band maximum (VBM) is majorly composed of V-*dz^2^* orbitals. On the other hand, this orbital composition becomes reverse for pristine VSi_2_As_4_ and Janus VSiGeP_2_As_2_, i.e., V-*dz^2^* orbitals contribute to the CBM, while V-*d_xy_* and V-*d_x_^2^_−y_^2^* form the VBM.

**Figure 4:**
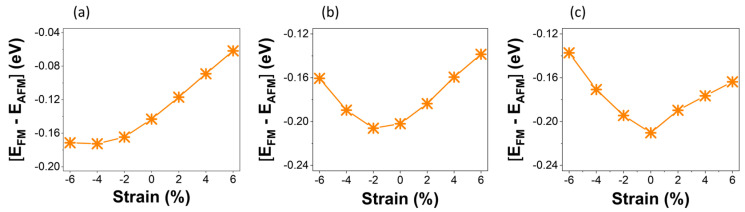
Strain dependence of energy differences between two magnetic configurations (FM and AFM) for (**a**) VSi_2_P_4_, (**b**) VSi_2_As_4_ and (**c**) VSiGeP_2_As_2_ Janus structures. The values of the Hubbard parameter used for the d-orbitals of V are U = 4 eV for VSi_2_P_4_, and 2 eV for VSi_2_As_4_ and VSiGeP_2_As_2_.

We studied the dependence of magnetic features of the VSi_2_Z_4_ and Janus VSiGeP_2_As_2_ on the biaxial strain. The energy difference between the FM and AFM configurations (E_FM_ − E_AFM_), which determines the magnetic ground for the material, is illustrated in Figure 4 as a function of compressive and tensile strains. All systems retain the FM orderings under different biaxial strains and do not show any phase transition from FM to AFM state with the applied strain. The strain, in this instance, is defined as follows:

$$\varepsilon =\left(\frac{a-a_{o}}{a_{o}}\right)\times 100\%$$

Here, ‘a_o_’ designates the lattice constant at a strainless state, and ‘a’ represents the strained lattice constant. The exchange parameter ‘J’, by taking into account the nearest neighbor exchange interactions, can be written as [28]:

$$J=-\left(\frac{E_{\mathrm{FM}}-E_{\mathrm{AFM}}}{6{\left|\vec{S}\right|}^{2}}\right)$$

where $\left|\vec{S}\right|$ = ½, as the electronic configuration 3d^3^4s^2^ becomes 3d^1^ after losing four electrons. The energy differences between the FM and AFM alignments can be easily calculated using DFT ground state formalism, which can be used to compute the Heisenberg exchange parameter ‘J’. The large value of ‘J’, together with the magnetocrystalline anisotropy, will produce a large critical temperature.

**Figure 5:**
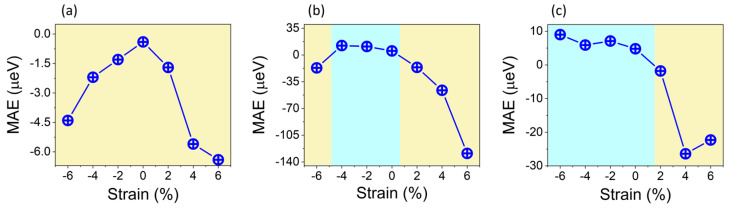
MAE as a function of biaxial strain calculated for two magnetic configurations ([001], [100]) (**a**) VSi_2_P_4_, (**b**) VSi_2_As_4_ and (**c**) VSiGeP_2_As_2_ Janus structures. Shaded regions indicate the modulation of the easy axis. The brown region is for an out-of-plane easy axis, while the cyan region indicates an in-plane easy axis. The values of the Hubbard parameter used for the d-orbitals of V are U = 4 eV for VSi_2_P_4_, and 2 eV for VSi_2_As_4_ and VSiGeP_2_As_2_.

The magnetic anisotropy energy (MAE) is used to determine the easy axis for magnetization direction. It is defined as the energy difference between the out-of-plane and in-plane spin alignments, i.e., MAE = E_⊥_ − E_||_. Consequently, a negative MAE will indicate an out-of-plane easy axis (perpendicular direction for magnetization), while positive values of MAE will indicate an in-plane easy axis (magnetization parallel to the plane direction). The MAE is originated because of the reliance of magnetic attributes on a specific crystallographic direction. Classically, dipole–dipole interactions are believed to be the origin of MAE, nonetheless quantum mechanically, the main cause lies in SOC [29]. For that reason, SOC effects should be considered in the evaluation of MAE. Thus, non-collinear calculations with SOC considered are carried out to evaluate the total energies (E_⊥_, E_||_) for the corresponding magnetization directions. We found MAE values of −4 μeV for VSi_2_P_4_ and 53 μeV in VSi_2_As_4_, indicating out-of-plane and in-plane magnetizations, respectively. Similarly, an in-plane magnetization is confirmed in ViSiGeP_2_As_2_ with an MAE value of 48 μeV. The direction of magnetization is essential to attain spontaneous valley polarization [14]. The effect of biaxial strain on MAE for all the monolayer systems is presented in Figure 5. One can see how the MAE is influenced by the tensile and compressive strains. For VSi_2_P_4_, the MAE decreases in either strain direction, with persistent out-of-plane easy axis for magnetization, as shown in Figure 5a. On the other hand, the in-plane easy axis in VSi_2_As_4_ is found tunable; it can be transformed to out-of-plane direction by applying some critical tensile or compressive strains, as indicated in Figure 5b. Likewise, an out-of-plane magnetization can be achieved in the Janus ViSiGeP_2_As_2_ monolayer at ε = 1.5%, as shown in Figure 5c. Shaded regions show the tuning of easy axis for the magnetization direction.

**Figure 6:**
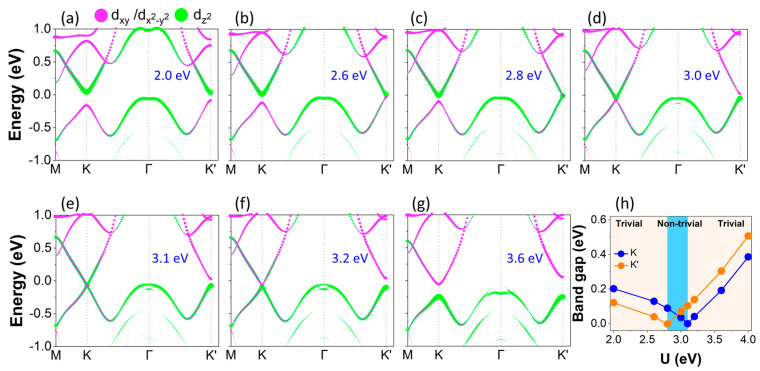
(**a**–**g**) Orbitally-resolved electronic band structures of the Janus VSiGeP_2_As_2_ monolayer with PBE + U and SOC included under different Hubbard parameter values U. The size of the colored dot is proportional to the weight of the corresponding orbitals. (**h**) Band gaps for the two K/K′ valleys.

**Figure 7:**
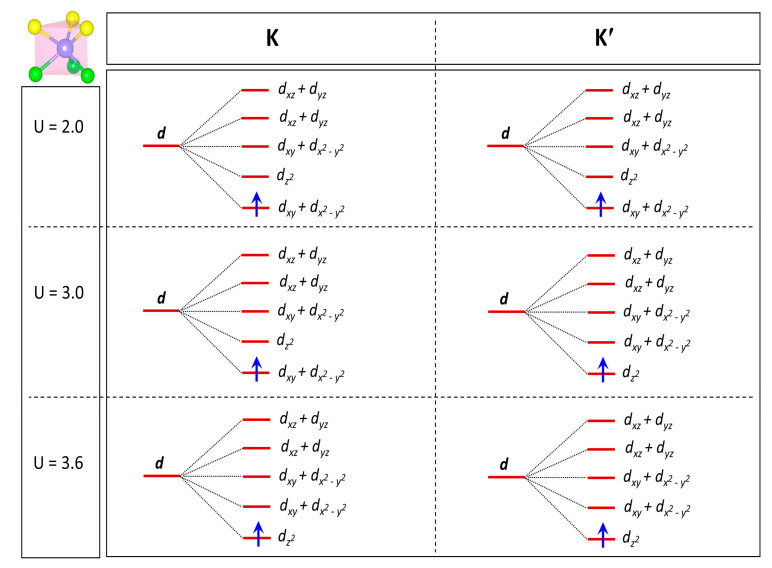
A schematic for the evolution of d orbitals of the spin up-subsector as a function of Hubbard parameter U for the Janus VSiGeP_2_As_2_ monolayer at K/K′ valleys. At U = 2 eV and U = 3.6 eV, the system is in the trivial ferrovalley insulating phase, while at U = 3 eV, it is in the topological phase.

Next, we show the electronic band structures of the Janus ViSiGeP_2_As_2_ monolayer by varying onsite Coulomb interaction known as the Hubbard parameter ‘U’, and by taking the SOC effect in consideration. Clearly, the CBM at the K/K′ valleys is made up of V-*dz^2^* orbitals when U = 2 eV is in the strain-free state, whereas the VBM is composed of V-*d_xy_* and V-*d_x_^2^_−y_^2^* states. Upon increasing the Hubbard parameter ‘U’, the V-*dz^2^* orbitals come down in energy, while the *d_xy_*/*d_x_^2^_−y_^2^* states go up in energy. When U reaches 2.8 eV, the system becomes gapless at the K′ point, although gapped at the K valley. The gapless nature of the band structure at K′ displays Weyl-like linear dispersion. Further raising U, the electronic band gap becomes smaller and smaller at the K valley. Conversely, at the K′ valley the band gap opens again with a band inversion exchanging the orbital contributions of the valence and conduction bands as compared to the band structure at U = 2 eV. Consequently, a topological phase transition occurs between U = 2.8 and U = 3.1 eV, leading to the emergence of the quantum anomalous Hall phase [5]. At U = 3.1 eV, the band gap closes at the K point and starts to reopen at 3.2 eV, with another band inversion achieved at the K valley. At U = 3.2 eV, we have a band inversion at both K and K′; as a result, the Janus structure is restored to the trivial ferrovalley insulating phase. The orbitally-projected band structure at U = 3.6 eV complies with all these behaviors. The evolution of band gaps and topological phases as a function of the electronic correlation at both K/K′ valleys is summarized in Figure 6h. As indicated, the trend of band gaps at the two valleys is quite similar; they begin to diminish, then reach zero, and finally they reopen by increasing U. As the band gap is smaller at K′ than at K valley (when U = 2 eV), the critical Hubbard parameter U necessary for closing the band gap is not similar; it is U = 2.8 eV and 3.1 eV, respectively. While usually the Coulomb repulsion kills the topological properties, in this case the Coulomb repulsion is necessary to observe the topological phase. Additionally, the range of U where the topological phase appears is between 2.8 and 3.1 eV, which is a realistic physical range for the Coulomb repulsion of 3d electrons. Moreover, the orbital characters at the K/K′ points of the Brillouin zone are investigated, as shown in schematic Figure 7, revealing the splitting of the energy levels of d orbitals in a trigonal prismatic crystal field environment. Here, only the middle layer containing V ions is displayed as the nonmagnetic top and bottom layers of these monolayers do not contribute to the spin density distribution.

## 4. Conclusions

In conclusion, based on first principles calculations, we present a detailed and comprehensive study of pristine VSi_2_Z_4_ (Z = P, As) and Janus VSiGeP_2_As_2_ monolayers. In the Janus phase, the compound shows breaking of the mirror symmetry, which is equivalent to having an electric field, and the system can be piezoelectric. After exploring their structural stability through ground state energies and phononic spectra, the electronic, magnetic and topological features were investigated. It was observed that these structures exhibit ground-state ferromagnetic ordering that persists at any tensile and compressive strains. In addition, VSi_2_P_4_ shows −4 μeV MAE with out-of-plane easy axis, which increases with the atomic number of pnictogens; for instance, in VSi_2_As_4_ the MAE increases dramatically to 53 μeV with in-plane magnetization direction. Likewise, an in-plane magnetization is established in VSiGeP_2_As_2_ with an MAE value of 48 μeV. In addition, we analyzed the effect of strain on the magnetic properties such as MAE, which revealed strong dependence on the biaxial strain.

We investigated how the topology of VSiGeP_2_As_2_ evolves as a function of the Coulomb interaction, and we observed the topological phase in the physical range of Hubbard U for 3d electrons. Our analysis of these emerging pristine and Janus-phased magnetic semiconductors opens prospects for studying the interplay between magnetism and topology in two-dimensional materials.

## Figures and Tables

**Table 1:** Optimized lattice constants a_o_, energy differences between the FM and AFM alignments and the easy axis for the magnetization.

Material	a_o_ (Å)	[E_FM_ − E_AFM_] (eV)	Easy Axis
VSi_2_P_4_	3.448	−0.143	Out-of-plane
VSi_2_As_4_	3.592	−0.202	In-plane
VSiGeP_2_As_2_	3.562	−0.210	In-plane

## Data Availability

The data that support the findings of this study are available on request from the corresponding author.
